# Mitochondrial metabolic plasticity in cancer: hybrid bioenergetic states and translational targeting in surgical oncology

**DOI:** 10.3389/ebm.2026.11244

**Published:** 2026-09-28

**Authors:** Debdeep Chattopadhyay, Ramya Lakshmi Rajendran, Nida Mubin, Durva Tendulkar, Thoudam Debraj Singh, Subhrojyoti Ghosh, Saurabh Kumar Jha, Prakash Gangadaran, Byeong-Cheol Ahn

**Affiliations:** 1 Post-Graduate & Research Department of Biotechnology, St. Xavier’s College (Autonomous), Kolkata, West Bengal, India; 2 Department of Nuclear Medicine, School of Medicine, Kyungpook National University, Daegu, Republic of Korea; 3 Cardiovascular Research Institute, Kyungpook National University, Daegu, Republic of Korea; 4 BK21 FOUR KNU Convergence Educational Program of Biomedical Sciences for Creative Future Talents, School of Medicine, Kyungpook National University, Daegu, Republic of Korea; 5 Division of Hematology and Oncology, Case Western Reserve University School of Medicine, Cleveland, OH, United States; 6 Department of Applied Microbiology, Vellore Institute of Technology, Vellore, India; 7 Department of Medical Oncology Laboratory, All India Institute of Medical Sciences (AIIMS), New Delhi, India; 8 Independant Researcher, Kolkata, India; 9 Department of Zoology, Kalindi College, University of Delhi, New Delhi, India; 10 Department of Biotechnology Engineering and Food Technology, Chandigarh University, Mohali, India; 11 Department of Nuclear Medicine, Kyungpook National University Hospital, Daegu, Republic of Korea

**Keywords:** biomarkers, cancer metabolism, mitochondria, perioperative care, surgical oncology, therapeutic targeting

## Abstract

Mitochondrial metabolism is increasingly recognized as an active determinant of tumor growth and cancer-cell adaptation rather than solely a source of cellular energy. Mitochondrial metabolism supports tumor anabolism, while mitochondrial DNA (mtDNA) alterations can affect respiratory chain function, redox homeostasis, and adaptation to changing tumor environments. At the same time, tumor metabolism is heterogeneous and flexible and cannot be explained by a uniform Warburg model. Tumor cells may use glycolysis, oxidative phosphorylation (OXPHOS), or hybrid glycolysis/OXPHOS states, with the relative contribution of these pathways varying across tumor types, tumor regions, microenvironmental conditions, and therapeutic pressure. This metabolic flexibility may contribute to tumor progression, metastasis, and treatment resistance. In this review, we examine mitochondrial metabolic phenotypes in cancer and their relevance to therapeutic targeting and surgical oncology. We discuss OXPHOS-dependent tumor states, mitochondrial DNA mutations, mitochondrial dynamics, reactive oxygen species, mitochondrial membrane potential, and mitochondrial regulation of intrinsic apoptosis. We further evaluate therapeutic strategies targeting these vulnerabilities, including OXPHOS inhibitors, BCL-2 family inhibitors, ROS modulators, mitochondrial dynamics modulators, mitochondrial genome-editing approaches, and emerging mitochondria-directed immunotherapies. Clinical evidence indicates that broad mitochondrial-targeted interventions may be limited by toxicity, narrow therapeutic windows, limited efficacy, or lack of benefit in unselected populations, supporting the need for metabolic phenotype classification, biomarker-guided patient selection, and rational combination strategies. Particular emphasis is placed on the potential integration of mitochondrial targeting with surgical oncology. Metabolic heterogeneity within tumors and the tumor microenvironment may influence treatment response, residual disease, and recurrence. Emerging approaches such as mass spectrometry imaging, single-cell sequencing, and spatial transcriptomic analysis may allow metabolic features to be mapped within anatomically defined regions of surgical specimens and linked to specific malignant, stromal, and immune-cell populations. Such approaches may help identify OXPHOS-dependent, glycolytic, and metabolically flexible tumor populations and provide clinically relevant biomarkers for treatment selection and monitoring. Overall, mitochondrial vulnerabilities represent a context-dependent therapeutic opportunity rather than a universal metabolic target. Better characterization of mtDNA alterations, metabolic plasticity, spatial heterogeneity, and tumor-specific metabolic dependencies will be important for developing biomarker-guided mitochondrial therapies and for determining how these interventions can be safely integrated into neoadjuvant, adjuvant, and perioperative cancer care.

## Impact statement

Mitochondrial reprogramming sustains cancer metabolism, survival, and resistance through the Warburg effect and altered oxidative phosphorylation. Dysregulated mitochondrial dynamics, DNA mutations, and ROS imbalance drive tumor progression and metastasis. Targeting mitochondrial dysfunctions offers novel therapeutic strategies to overcome cancer resistance and improve treatment efficacy.

## Introduction

Mitochondria are increasingly positioned as active determinants of tumor growth and phenotype rather than passive bioenergetic remnants, with evidence in humans and mice supporting that mitochondrial metabolism is necessary for tumor growth and supports tumor anabolism by providing metabolites for macromolecule synthesis and related cancer-maintaining programs [1]. Consistent with this, mitochondria and mtDNAs are described as essential for cancer cell growth, and mtDNA mutations across cancers are described as altering mitochondrial metabolism and permitting adaptation to changing environments [2]. These observations provide a mechanistic rationale for why mitochondria have become attractive targets for cancer therapy and why multiple clinical trials have been launched to test the efficacy of inhibiting mitochondrial metabolism in cancer treatment contexts [3, 4].

Tumor metabolism is not adequately explained by a uniform “Warburg-only” model [5]. Glucose utilization and carbon sources are described as more heterogeneous than initially appreciated [6–8], and recent work emphasizes heterogeneity and flexibility of metabolism between tumors and within distinct regions of solid tumors rather than only stereotyped cell-autonomous glycolysis [6, 9, 10]. Tumor cells are described as having a dual capacity for glycolytic and OXPHOS metabolism [9, 11–13], and emerging evidence supports hybrid glycolysis/OXPHOS states in which both pathways are used for energy production and biomass synthesis [11, 14, 15]. The ability to switch between glycolysis and OXPHOS in response to environmental cues and therapeutic pressures is repeatedly highlighted as a hallmark of metabolic plasticity that complicates single-pathway interventions.

Hybrid glycolysis/OXPHOS phenotypes are described as facilitating metabolic plasticity and as being specifically associated with metastasis and therapy resistance [10, 15, 16], linking metabolic state to clinically consequential behaviours that influence recurrence risk after multimodal management, including surgery. Intratumoral heterogeneity can include coexisting populations with distinct metabolic programs and such populations are described as being able to reverse their energetic control by switching between glycolysis and oxygen-based processes [9], reinforcing a phenotype-aware view of surgical specimens and treatment resistance biology.

These concepts motivate a reframed translational agenda that is compatible with surgical care pathways. First, analyses support strategies aiming to classify tumors by metabolic phenotype in order to therapeutically target tumor-specific vulnerabilities [17], creating a biomarker-first rationale for stratification in neoadjuvant and adjuvant settings. Second, mitochondria can generate immune-modulating injury signals: mtDNA release into cytosolic and extracellular compartments is described as activating pattern recognition receptors and innate immune responses, including cGAS–STING, TLR9, and inflammasome formation [18], establishing a plausible mechanism by which tissue injury and cellular stress might shape systemic inflammatory signaling. Third, because OXPHOS inhibitors are described as having limited efficiency in monotherapy and remaining most interesting in combinations with conventional therapies [3, 4]. Surgical oncology integration is best conceptualized as biomarker-guided combinations evaluated in pragmatic peri-treatment windows rather than as universally applicable “metabolic drugs”.

Accordingly, this review synthesizes (i) evidence for metabolic heterogeneity, dual-capacity metabolism, and hybrid glycolysis/OXPHOS states as determinants of progression and resistance, (ii) mechanistic anchors for mitochondrial dysfunction in cancer that are relevant to translational stratification, including mtDNA alterations and mitochondrial dynamics, and (iii) clinical and translational lessons from mitochondria-targeted therapy development, emphasizing both promise and limitations in trial outcomes and feasibility. Mitochondria play central roles in cellular metabolism, ATP production, biosynthetic pathways, and the regulation of apoptosis (Figure 1).

**FIGURE 1:**
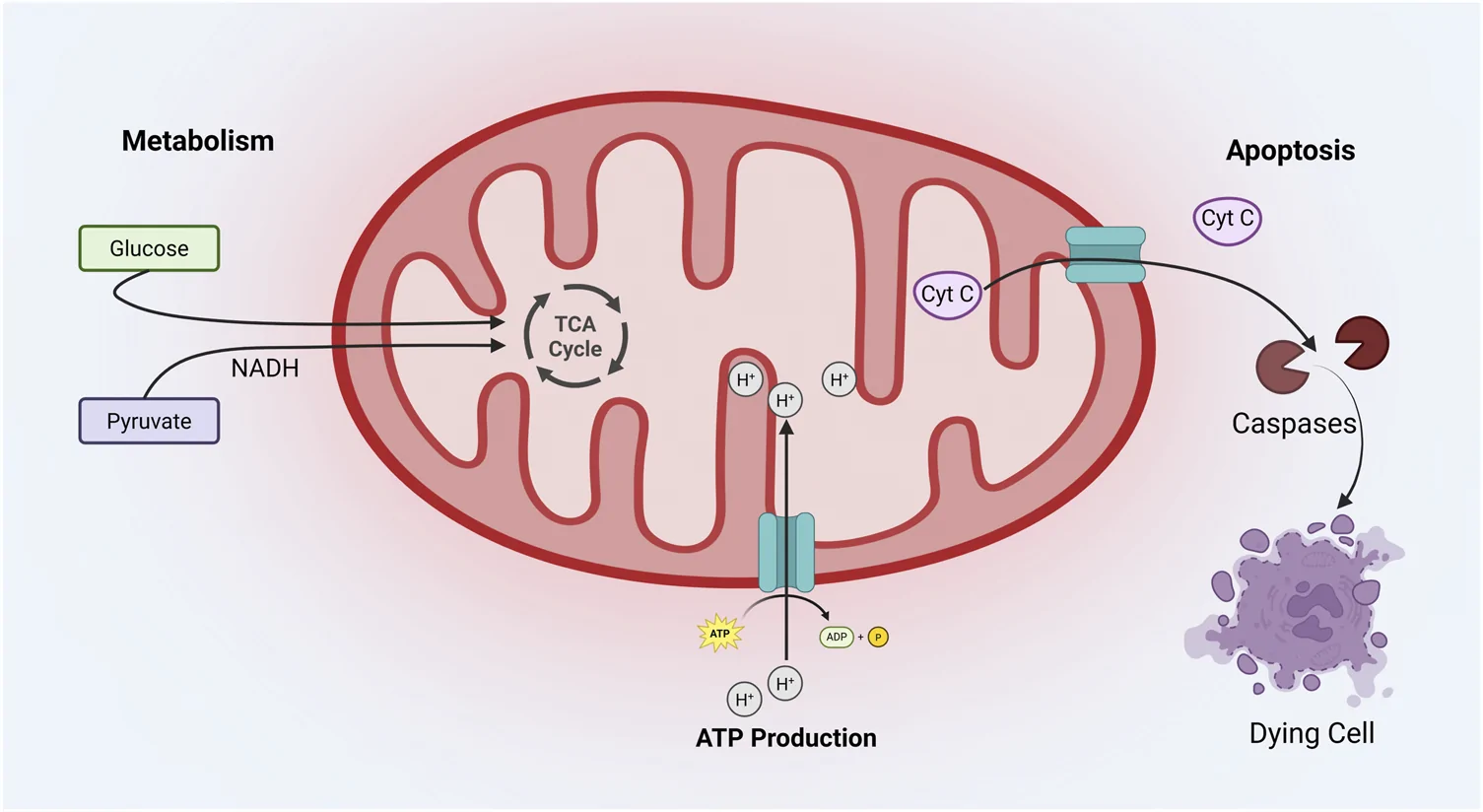
Various roles of mitochondria, from cellular metabolism to energy production to apoptosis. Created in BioRender. Gangadaran, P. (2026) https://BioRender.com/ub92a2m.

## Mitochondrial metabolic plasticity in surgical oncology: mechanisms, therapeutic targeting, and clinical integration

### Methods

This review searched PubMed/MEDLINE, Embase, and Web of Science for relevant publications from 2020 to 2026, supplemented by selected seminal studies published before 2020 and relevant studies published in 2026. Search terms covered mitochondrial metabolism, OXPHOS, metabolic plasticity, mitochondrial dysfunction, cancer, therapeutic targeting, biomarkers, and surgical oncology. Original experimental, translational, and clinical studies, together with relevant reviews, were included.

### Mitochondrial metabolic phenotypes in surgical oncology

Tumor metabolic phenotypes are described as heterogeneous across and within solid tumors, with important consequences for therapy selection, resistance, and biomarker-driven translational strategies that can be aligned with multimodal cancer care including surgery.

### Beyond the warburg effect

The Warburg effect describes the propensity of tumor cells to preferentially metabolize glucose through glycolysis even in the presence of oxygen, rather than relying on oxidative phosphorylation. However, glucose utilization and carbon sources in tumors are described as much more heterogeneous than initially thought, and recent work emphasizes heterogeneity and flexibility between tumors and within distinct regions of solid tumors [5].

In this contemporary framing, tumor cells are described as having a dual capacity for glycolytic and OXPHOS metabolism, and many cancer cells are described as being able to oxidize glucose via OXPHOS in fully functioning mitochondria, supporting a view in which mitochondrial metabolism remains functionally important in many cancers. Cancer cells are also described as being able to switch between glycolysis and OXPHOS and back under different environmental conditions, and more generally as having the ability to switch between glycolysis and OXPHOS in response to environmental cues and therapeutic pressures, emphasizing plasticity as a resistance-relevant property [12, 19].

Beyond “switching,” emerging evidence supports a hybrid glycolysis/OXPHOS phenotype in which both glycolysis and OXPHOS can be utilized for energy production and biomass synthesis, and this hybrid phenotype is described as facilitating metabolic plasticity and as being associated with metastasis and therapy resistance. Systems-level approaches further support that cancer cells can access a hybrid state with both metabolic modes coexisting, and gene-signature approaches have been proposed to quantify glycolysis and OXPHOS activity via AMPK and HIF-1 downstream genes, providing conceptual tools for metabolic phenotyping [13]. Metabolic changes during metastasis are not simply a result of increased glycolysis. While glycolysis can support tumor growth, invasion, and adaptation in some settings, metastatic cells can also rely on mitochondrial respiration and OXPHOS during dissemination and colonization of distant organs. These metabolic differences may depend on the specific metastatic site, as well as local oxygen and nutrient availability and interactions with the surrounding tumor microenvironment. Therefore, the relationship between glycolysis and OXPHOS during metastasis is more complex than a simple switch from one pathway to the other [20–22]. In this context, the hybrid glycolysis/OXPHOS phenotype may represent one form of metabolic plasticity rather than a common feature of all metastatic tumors. Some tumor cells may use both pathways and shift between them in response to changes in their environment or treatment, while other tumors or metastatic lesions may depend more strongly on either glycolysis or mitochondrial respiration [11, 23]. Recognizing these differences is important for understanding metabolic vulnerabilities and for developing therapies that target mitochondrial metabolism [24, 25].

Mechanistically, oncogene- and hypoxia-linked programs can converge on glycolytic effectors, exemplified by the observation that HK2, PKM2, and LDHA are targets of both c-Myc and HIFs, and synthesis work describes dysregulated glycometabolism as a hallmark driven by oncogenes such as c-Myc and K-ras and linked to plasticity supporting progression, metastasis, treatment resistance, and recurrence [26, 27]. Metabolic reprogramming is controlled by several oncogenic and stress-responsive pathways rather than by glycolysis or OXPHOS alone. Under hypoxic conditions, HIF-1α promotes expression of glycolytic genes and supports adaptation to limited oxygen availability, whereas c-Myc can increase glucose and glutamine utilization and support the biosynthetic demands of proliferating tumor cells. PI3K–AKT–mTOR signaling also promotes nutrient uptake and anabolic metabolism, while AMPK responds to energy stress and helps maintain cellular energy balance [7]. In contrast, PGC-1α has been linked to mitochondrial biogenesis and increased OXPHOS in specific tumor contexts. These pathways can interact and produce different metabolic states within the same tumor, providing a mechanistic basis for the metabolic heterogeneity and flexibility described above.

### OXPHOS-dependent tumor subtypes

Within metabolic heterogeneity, specific tumor contexts and subpopulations are described as highly dependent on OXPHOS, providing a rationale for phenotype-aware targeting of mitochondrial respiration, where switching to other metabolic pathways is constrained. Preclinical development programs for complex I inhibition have highlighted multiple tumor types with high OXPHOS dependence, including AML and subsets of lymphoma, glioblastoma, triple-negative breast cancer, melanoma, and pancreatic ductal adenocarcinoma [28].

Melanoma is a canonical example, as PGC-1α-dependent mitochondrial oxidative metabolism is described as essential for maintaining growth and survival of a subset of human melanomas, supporting a biologically grounded metabolic subtype that may be relevant for stratification in translational studies and in the interpretation of surgical specimens and recurrence biology. More broadly, work emphasizing that cancer cells do not generate energy only through aerobic glycolysis but that some subtypes strongly rely on OXPHOS reinforces the need for phenotype classification rather than assuming uniform glycolytic dependence across cancers [29, 30]. The Table 1 summarizes selected OXPHOS-relevant contexts and biomarker concepts that emerge directly from the cited evidence.

**TABLE 1:** OXPHOS-dependent tumor contexts and candidate stratification concepts.

Tumor context	Evidence of OXPHOS relevance	Candidate stratification concept
Melanoma (subset)	PGC-1α-dependent mitochondrial oxidative metabolism is essential for growth and survival of a subset of human melanomas	PGC-1α/OXPHOS-aligned transcriptional programs as a subtype concept
AML	OXPHOS dependence is highlighted in complex I inhibitor development programs and clinical testing contexts	Exploratory response biology endpoints including OCR/aspartate and gene-expression readouts are described as validated for use in clinical biology of response studies
PDAC (subset)	PDAC is highlighted among OXPHOS-dependent contexts in complex I inhibitor development programs	Metabolic phenotype classification is proposed as a strategy to target tumor-specific vulnerabilities (principle)
TNBC (subset)	OXPHOS dependence is highlighted in subsets of triple-negative breast cancer in complex I inhibitor programs	Exploratory response biology endpoints (OCR/aspartate, transcriptional readouts) used in OXPHOS inhibitor development (concept)
Glioblastoma (subset)	OXPHOS dependence is highlighted in subsets of glioblastoma in complex I inhibitor programs	Intratumoral metabolic heterogeneity provides a rationale for region- and subtype-selective vulnerabilities (concept)

### Reactive oxygen species in cancer

Mitochondrial redox biology is linked to cancer phenotypes through oxidative damage and immune-modulating danger signaling, and it is also positioned as a therapeutic axis via strategies that modulate ROS levels or mitochondrial stress outputs [31].

At the genomic level, mtDNA is described as not being protected by histones and thus as extremely vulnerable to oxidative damage generated in the matrix, providing a mechanistic link between oxidative stress, mtDNA alteration, and downstream metabolic rewiring in tumors. From a therapeutic standpoint, pancreatic cancer synthesis highlights that a promising strategy is to increase intracellular ROS to make cancer cells more vulnerable to oxidative stress–induced cell death, supporting continued interest in pro-oxidant approaches [32, 33].

Mitochondria-targeting organic sensitizers are described as modulating mitochondrial functions including membrane potential dynamics and ROS generation while releasing damage-associated molecular patterns that activate innate and adaptive immunity, linking redox and mitochondrial stress to immunogenic signaling outputs and reinforcing that mitochondria influence immune responses within the tumor microenvironment [34, 35].

### Mitochondrial DNA mutations

mtDNA mutations are described as being found in various cancers and as altering mitochondrial metabolism in ways that enhance tumorigenesis and permit adaptation to changing environments, supporting their relevance to both biology and biomarker considerations. mtDNA mutations are reported in genes for respiratory chain complexes (including complexes I, III, and IV), reinforcing the plausibility that mtDNA variation can shape OXPHOS-related phenotypes and redox stress [36]. Because mitochondrial respiration contributes to ATP production, biosynthetic processes, and redox regulation, alterations in mtDNA may affect several aspects of tumor-cell behavior rather than representing isolated genetic events.

The functional consequences of mtDNA mutations are also influenced by the proportion of mutant and wild-type mitochondrial genomes within a cell. This heteroplasmic state may contribute to differences in mitochondrial function between tumor cells and between regions of the same tumor. As a result, the presence of an mtDNA mutation alone may not predict its functional effect. The metabolic consequences may depend on the specific mutation, heteroplasmy level, cellular context, and interaction with nuclear-encoded mitochondrial pathways. These factors may contribute to the heterogeneous metabolic phenotypes observed within tumors [37].

Recent clinical findings also suggest that mitochondrial DNA content may be relevant to treatment selection. In a 2024 study of 308 patients with mismatch repair-deficient colorectal cancer, higher tumor mtDNA copy number (mtDNA-CN) was associated with better disease-free and overall survival. Patients with higher mtDNA-CN also appeared to derive greater benefit from adjuvant chemotherapy, including patients with stage II and stage III disease [38]. These findings are particularly relevant to surgical oncology because mtDNA-CN can be evaluated in resected tumor tissue and may provide additional information for postoperative treatment decisions. However, these observations still require prospective validation before mtDNA-CN can be used routinely to guide adjuvant chemotherapy [38].

Beyond its potential prognostic value, mtDNA-CN may provide additional information for postoperative treatment planning and could be considered alongside established clinicopathologic factors when selecting patients for adjuvant chemotherapy. However, current evidence supports mtDNA-CN as an investigational biomarker, and prospective studies are needed before it can be incorporated into routine postoperative treatment or surveillance protocols.

Given that mtDNA is highly vulnerable to oxidative damage in the matrix, oxidative stress provides a mechanistic basis for the accumulation of mutations, selection, and downstream effects on metabolism and adaptation in tumors. At the same time, the field emphasizes that functional studies, haplotype analysis, and extensive single-cell sequencing are necessary to understand the roles of individual mtDNA variations in tumor progression, underscoring that descriptive catalogs of mutations require functional integration to become clinically actionable [39].

Emerging preclinical approaches are beginning to exploit mtDNA variation therapeutically. mitochondria-penetrating conjugates has been investigated for selective of mtDNA variants, including approaches designed to reduce mutant mtDNA in heteroplasmic cells or induce apoptosis in cells with near-homoplasmic pathogenic mutations [40]. These strategies remain preclinical, and questions concerning specificity, delivery, heteroplasmy, off-target effects, and the selection of clinically relevant mtDNA variants require further investigation.

Overall, mtDNA alterations may contribute to the metabolic heterogeneity seen across cancers, but their clinical significance cannot be understood from mutation profiles alone. The functional effects of individual mutations and mtDNA-CN may vary between tumors and influence treatment response [36–38]. Recent clinical evidence suggests that mtDNA-CN may be useful for treatment stratification, particularly in surgically treated colorectal cancer, although further prospective studies are needed before it can be incorporated into routine clinical decision-making [38]. Combining mtDNA sequencing and mtDNA-CN analysis with metabolic measurements, single-cell approaches, and clinical data may help identify mitochondrial alterations that are relevant to prognosis and treatment.

### Mitochondrial dynamics

Mitochondrial morphology and dynamics are increasingly integrated into metabolic control circuits, linking mitochondrial network state to the balance between glycolysis and OXPHOS and thereby to cancer cell growth control mechanisms. Mechanistically, PKM2 is reported to bind MFN2 and thereby promote mitochondrial fusion and OXPHOS with concomitant attenuation of glycolysis, establishing a direct connection between a core glycolytic enzyme and fusion machinery [41].

Upstream signaling is also implicated: mTOR is reported to directly phosphorylate MFN2, and an mTOR–MFN2–PKM2 signaling axis is described as critical for the glycolysis/OXPHOS switch controlling cancer cell growth, with phosphorylation of MFN2 reported as affecting growth by promoting fusion and OXPHOS while suppressing glycolysis. These findings support the interpretation that mitochondrial “form” and metabolic “state” can be co-regulated, motivating interest in whether dynamics-related markers could serve as part of phenotype classification or as co-targets in combination strategies [18]. Multiple factors, including mtDNA mutations, oxidative stress, impaired mitophagy, altered biogenesis, and electron transport chain defects contribute to mitochondrial dysfunction in cancer (Figure 2).

**FIGURE 2:**
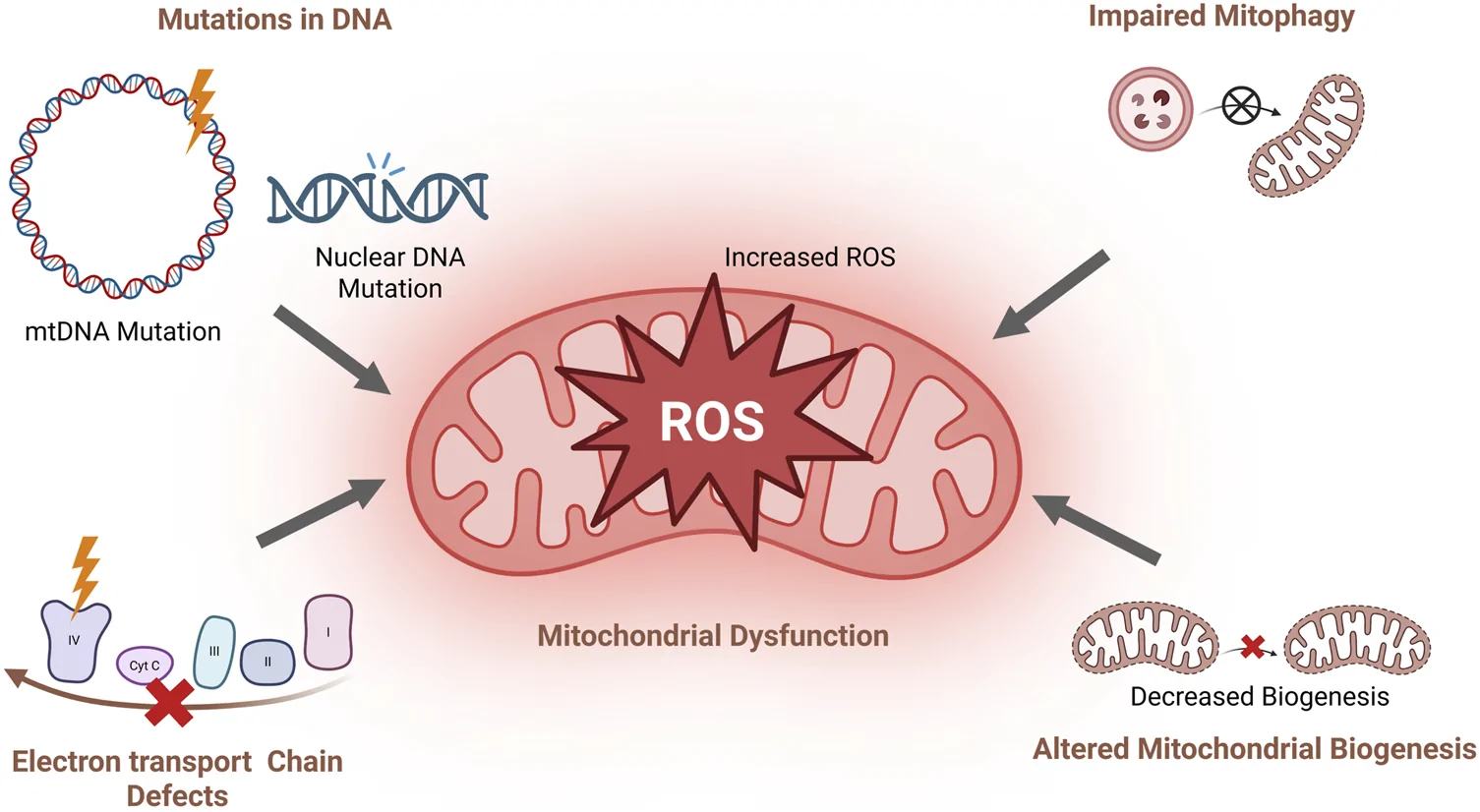
Various factors contributing to mitochondrial dysfunction, including mutations in mitochondrial or genomic DNA, impaired mitophagy, increased ROS, altered mitochondrial biogenesis, and defects in the electron transport chain (ETC). Created in BioRender. Gangadaran, P. (2026) https://BioRender.com/rq57129.

On the intervention side, a phase I clinical trial of the DRP1 inhibitor mdivi-1 is described as reducing intestinal mucosal inflammation and repairing barrier function by inhibiting excessive mitochondrial fission, but this evidence is described in a non-oncologic inflammatory disease context and does not establish clinical anticancer efficacy in surgical oncology settings. More broadly, translational efforts targeting mitochondria are described as being constrained by resistance mechanisms, safety, toxicity, and mitochondrial heterogeneity, reinforcing that dynamics modulation will require careful evaluation for tumor selectivity versus host toxicity in clinically relevant contexts [42].

### Apoptosis evasion

Mitochondria-directed intrinsic apoptosis remains central to many anticancer strategies, and mitochondrial perturbation can induce intrinsic apoptosis pathways in tumor cells. Preclinical tumor models, including emerging gene-therapy approaches, have demonstrated cytotoxic effects that are mediated predominantly through mitochondrial-dependent intrinsic apoptosis [43]. Venetoclax is described as a BH3 mimetic that selectively antagonizes antiapoptotic BCL-2 and shows particularly significant treatment effects in hematologic malignancies, however, its efficacy in solid tumors has been less established. In the randomized phase II VERONICA trial of ER-positive, HER2-negative metastatic breast cancer, venetoclax combined with fulvestrant did not significantly improve clinical benefit rate or progression-free survival compared with fulvestrant alone, highlighting the importance of tumor context, biomarker selection, and rational combination strategies in solid tumors [44, 45]. The Bcl-2 family is a key targeting, regulatory axis within this pathway, with anti-apoptotic proteins such as BCL-2, BCL-xL, and MCL-1 regulating mitochondrial apoptotic priming and the commitment to mitochondrial outer-membrane permeabilization. Dysregulation of this balance can promote evasion of apoptosis and contribute to therapeutic resistance. Consequently, pharmacologic targeting of BCL-2-family proteins represents an important strategy for restoring apoptotic susceptibility, with clinically mature evidence and translational challenges discussed in Section 5.2; [46].

### Peri-treatment mitochondrial biology

Peri-treatment translation in surgical oncology can be anchored to mechanisms connecting tissue stress, inflammation, metabolic adaptation, and immune remodelling, and mitochondrial pathways provide a plausible convergence point because mitochondrial stress can generate DAMPs and reshape immune signaling.

### Mitochondrial modulation and immunity

Mitochondria are described as influencing immune responses within the tumor microenvironment, providing a conceptual basis for peri-treatment windows in which immune remodelling could affect residual disease control in multimodal therapy contexts. Mitochondria-targeting organic sensitizers are described as modulating mitochondrial functions (including membrane potential dynamics and ROS generation) and releasing DAMPs that potently activate innate and adaptive immunity, linking mitochondrial perturbation to immune activation in translational therapy concepts [46].

Mitochondrial integrity is crucial for antitumor T-cell function because oxidative phosphorylation and complex III-derived mitochondrial ROS support NFAT signaling, IL-2 production, and antigen-specific T-cell expansion [47]. Within the tumor microenvironment, persistent antigen stimulation, hypoxia, and nutrient deprivation suppress PGC-1α-dependent mitochondrial biogenesis, reduce mitochondrial mass and respiratory capacity, and induce excessive ROS accumulation in CD8^+^ tumor-infiltrating T cells [48, 49]. These changes impair proliferation, cytokine production, cytotoxicity, and persistence, while promoting terminal exhaustion and diminished responsiveness to immunotherapy. Mitochondrial dynamics also influence T-cell fate, with fused mitochondrial networks supporting oxidative metabolism and durable memory formation [50]. Importantly, restoring mitochondrial fitness through PGC-1α activation or mitochondrial pyruvate carrier-dependent oxidative phosphorylation can reinvigorate dysfunctional or terminally exhausted CD8^+^ T cells and enhance antitumor activity [48, 51]. Therefore, mitochondria-targeted therapies should selectively disrupt tumor metabolism while preserving or enhancing mitochondrial function of T cells to support effective immune surveillance and improve responses to immune checkpoint blockades or adoptive cell therapies.

At the same time, clinical translation of mitochondria-targeted sensitizers is described as facing challenges including variable mitochondrial targeting efficiency, systemic toxicity risks, insufficient long-term biocompatibility evaluation, and reliance on simplified tumor models that inadequately reflect clinical heterogeneity and spatiotemporal dynamics of mitochondrial damage–immune remodeling interactions, emphasizing feasibility constraints relevant to peri-treatment or perioperative deployment [52].

### Mitochondrial DAMPs

When mtDNA is released into the cytoplasm and extracellular environment, it is described as activating pattern recognition receptors and innate immune responses including the cGAS–STING pathway, TLR9 receptors, and inflammasome formation, establishing a mechanistic bridge between mitochondrial injury and systemic inflammatory signaling.

### Oxygen consumption and treatment sensitivity

Solid tumors are described as metabolically heterogeneous, comprising oxidative and glycolytic cancer and host cells, implying that interventions that alter mitochondrial oxygen consumption can have spatially selective effects on treatment sensitivity in heterogeneous tissue microenvironments. In this context, the mitochondria-targeted antioxidant MitoQ is reported to dose-dependently reduce cancer cell respiration, consistent with a mechanistic strategy to alter oxygen consumption and thereby potentially modify oxygenation-dependent treatment response in selected settings [53].

Preclinical work reports that MitoQ radiosensitizes MDA-MB-231 human breast tumors in mice, and authors conclude that MitoQ can radiosensitize MDA-MB-231 but not MCF7 cancer cells, emphasizing context dependence consistent with metabolic heterogeneity and motivating biomarker-linked development strategies rather than uniform deployment [54, 55].

Separately, preclinical breast cancer models report that local recurrence and metastatic dissemination can be prevented by MitoQ, and oral delivery is reported to be effective at nanomolar concentrations at which nonmalignant cells were spared, supporting the concept that mitochondrial redox modulation can have selective antitumor effects in specific contexts. To support translational stratification, a robust signature of response to MitoQ is described as defined and applicable to bulk primary tumor biopsies, aligning with a biomarker-first approach for future clinical testing [56].

### Early clinical testing signals

ClinicalTrials.gov records indicate that mitochondria-related interventions are entering early clinical testing in advanced solid tumors, including a phase I dose-escalation study using subcutaneous injections of an engineered mitochondrial vaccine in advanced solid tumors. The registry specifies primary and secondary outcome measures including objective response rate, which situates such trials within standard oncology endpoint frameworks while not providing outcomes data itself.

### Mitochondria-targeted therapeutics

Mitochondria-targeted therapeutics are pursued because actively functional mitochondria are described as playing critical roles in tumorigenesis, metastasis, cancer stemness, and therapy resistance, and because mitochondrial metabolism is described as active and necessary for tumor growth and tumor anabolism in multiple contexts. However, metabolic heterogeneity and plasticity—encompassing dual-capacity metabolism and hybrid states—create strong rationale for biomarker-guided combinations rather than empiric monotherapy across unselected surgical populations.

Current therapeutic strategies targeting mitochondrial vulnerabilities in cancer include metabolic inhibitors, ROS modulators, regulators of mitochondrial membrane potential, modulators of mitochondrial dynamics, mitochondrial genome-editing approaches, and emerging mitochondria-directed immunotherapies (Figure 3).

**FIGURE 3:**
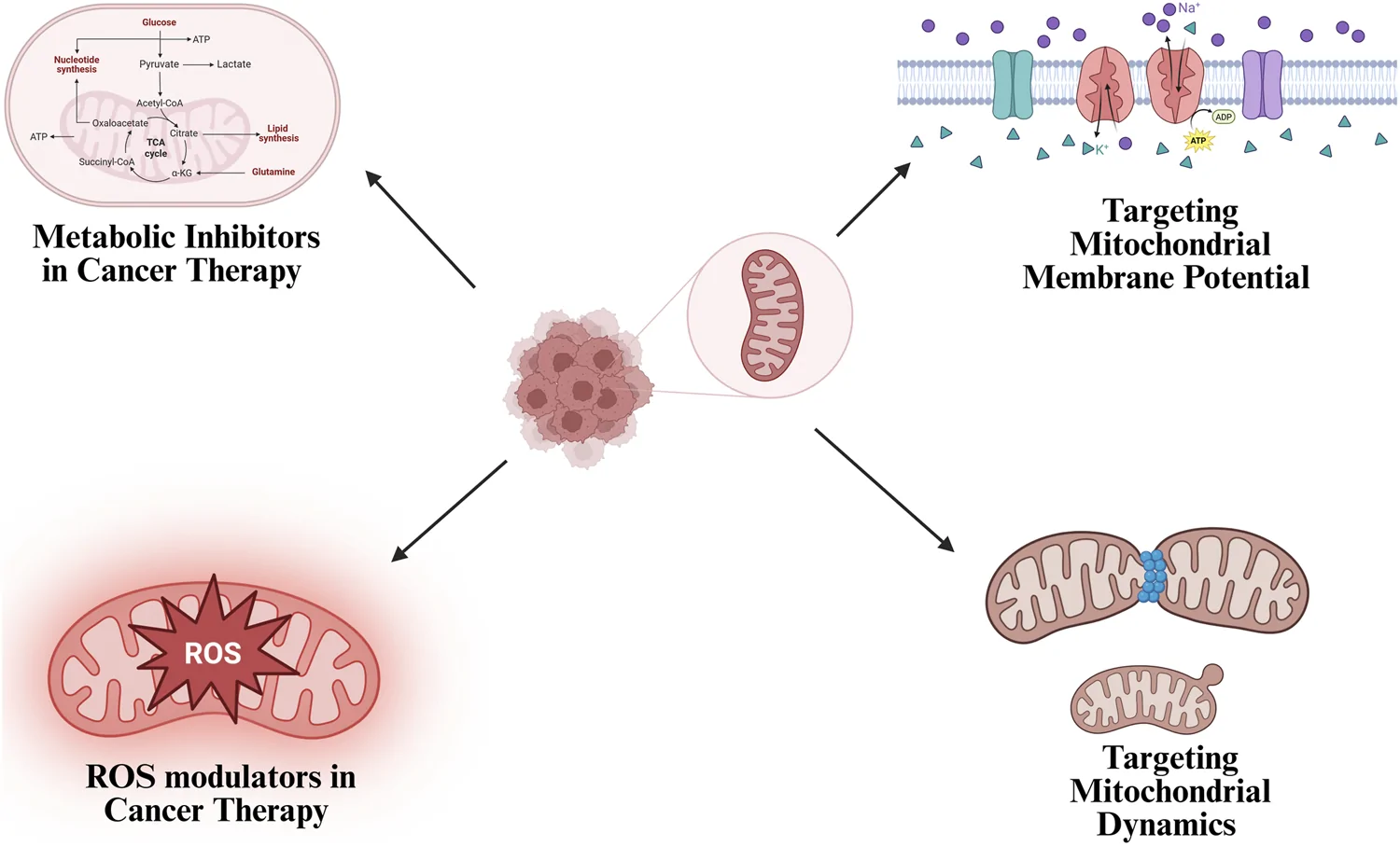
Therapeutic strategies targeting mitochondria in cancer, including metabolic inhibitors, reactive oxygen species (ROS) modulators, regulators of mitochondrial membrane potential, and modulators of mitochondrial dynamics. Created in BioRender. Gangadaran, P. (2026) https://BioRender.com/19pcvsm.

### OXPHOS inhibitors

Metformin is described as another drug targeting respiration in cancer-related metabolic frameworks, but randomized evidence in early breast cancer indicates that broad adjuvant use is not supported by clinical outcomes in unselected populations. In the MA.32 trial, the addition of metformin versus placebo to standard breast cancer treatment did not significantly improve invasive disease–free survival, and grade 3 nonhematological toxic events occurred more frequently with metformin than placebo. In MA.32 adherence analyses among hormone receptor–positive patients, non-adherence to study drug is reported as common and higher in patients receiving metformin than placebo, illustrating a feasibility constraint relevant to any long-duration peri-treatment metabolic intervention strategy [4, 57].

MA.32 also illustrates that measurable biomarker modulation does not necessarily translate into improved survival endpoints in unselected populations. At 6 months, CA 15-3 was statistically significantly reduced in metformin versus placebo arms, and this reduction was reported as independent of tumor characteristics and perioperative systemic therapy; additionally, median estradiol decreased on metformin versus placebo in analyses reported as independent of BMI, which suggests measurable physiologic effects relevant to translational “window” endpoints even when primary efficacy endpoints are negative [58].

IACS-010759 is described as a potent, selective small-molecule inhibitor of mitochondrial complex I that advanced into phase I clinical trials in relapsed/refractory AML and solid tumors, providing a key case study for OXPHOS inhibition translation. Phase I results indicate a narrow therapeutic index with dose-limiting toxicities including elevated blood lactate and neurotoxicity that obstructed efforts to maintain target exposure, and consequently no recommended phase 2 dose was established, only modest target inhibition and limited antitumor activity were observed at tolerated doses, and the trials were discontinued. The same dataset reports that achieving and maintaining plasma exposures higher than those observed during the trials would have been needed for clinical benefit, and IACS-010759 administration increased extracellular lactate in responsive tumor cells, reinforcing both exposure limitations and metabolic adaptation signals relevant to future biomarker-guided trial designs [59]. Beyond metformin and IACS-010759, multiple OXPHOS inhibitors (including phenformin and IM156) are described as under evaluation, reflecting sustained interest in respiration targeting despite clinical and translational barriers highlighted by trial outcomes and metabolic plasticity considerations [60]. Another approach to targeting mitochondrial metabolism is inhibition of the tricarboxylic acid (TCA) cycle. Devimistat (CPI-613), a lipoate analog that targets pyruvate dehydrogenase and α-ketoglutarate dehydrogenase complexes, has been investigated in several cancers, particularly pancreatic cancer. In the phase III AVENGER 500 trial, however, adding devimistat to modified FOLFIRINOX did not improve overall survival or progression-free survival compared with FOLFIRINOX alone in patients with metastatic pancreatic cancer [61, 62]. These findings highlight both the clinical interest in TCA-cycle inhibition and the limitations of broadly targeting mitochondrial metabolism without appropriate patient selection or biomarkers [61].

### Bcl-2 family targeting

The BCL-2 family represents a clinically established therapeutic axis for targeting mitochondrial apoptotic priming. Anti-apoptotic proteins, including BCL-2, BCL-xL, and MCL-1, regulate mitochondrial outer-membrane permeabilization and determine the threshold for intrinsic apoptosis. Pharmacologic inhibition of these proteins can therefore lower the apoptotic threshold and restore susceptibility to cell death in tumors that retain dependence on specific anti-apoptotic pathways [63].

Venetoclax a selective BCL-2 inhibitor and BH3 mimetic, provides the most clinically mature example of this strategy. Its therapeutic activity is particularly well established in hematologic malignancies, including the phase 3 VIALE-A trial in which azacitidine plus venetoclax produced longer overall survival than azacitidine alone in previously untreated AML patients not eligible for intensive therapy. These findings provide clinical proof of principle that therapeutically manipulating mitochondrial apoptotic priming can produce substantial antitumor benefit [64, 65].

In contrast, translation of BCL-2 inhibition to solid tumors has been more challenging. Venetoclax monotherapy has demonstrated limited activity in several solid-tumor settings, highlighting the importance of tumor-specific apoptotic dependence and appropriate patient selection. In the cited endocrine-therapy combination study, venetoclax plus fulvestrant did not significantly improve clinical benefit rate or progression-free survival compared with the comparator arm [66]. In one example, the clinical benefit rate and progression-free survival were not significantly improved in a venetoclax plus fulvestrant arm, illustrating the challenge of extrapolating hematologic success to solid tumor settings without stronger biomarker logic [66].

Broader targeting of the BCL-2 family also presents a therapeutic window challenge. Navitoclax, which inhibits BCL-2, BCL-xL, and BCL-w, illustrates the toxicity associated with broader anti-apoptotic targeting, with thrombocytopenia representing a major dose-limiting concern in clinical development [67]. These observations highlight the importance of balancing apoptotic sensitization with normal-tissue toxicity when considering mitochondrial-targeted therapies.

This consideration is particularly relevant to surgical oncology. Venetoclax-associated adverse events, including tumor lysis syndrome, anemia, neutropenia, and infections, may affect the feasibility and timing of treatment around surgery [67]. Accordingly, future evaluation of BCL-2-family inhibitors in neoadjuvant or postoperative minimal-residual-disease settings should incorporate tumor-specific biomarkers, treatment sequencing, hematologic recovery, infection risk, and postoperative healing. Biomarker-guided combination strategies may ultimately provide a more rational approach than broad application of BCL-2 inhibition across unselected solid-tumor populations.

### Mitochondrial dynamics modulators

Mitochondrial dynamics remain conceptually attractive because they are mechanistically connected to metabolic state. PKM2 binding to MFN2 is reported to promote mitochondrial fusion and OXPHOS with attenuation of glycolysis, and an mTOR–MFN2–PKM2 signaling axis is described as critical for metabolic switching between glycolysis and OXPHOS in cancer cell growth control [41].

Therapeutic modulation of mitochondrial dynamics remains at an early stage of clinical translation. mdivi-1, a commonly used experimental inhibitor of mitochondrial fission, has been investigated clinically in a phase I study in an inflammatory disease setting rather than in oncology [18]. Therefore, available clinical evidence should not be interpreted as evidence of anticancer efficacy. Further investigation is required to determine whether modulation of mitochondrial fission or fusion can provide a therapeutically useful window in cancer while minimizing toxicity and accounting for tumor-specific mitochondrial heterogeneity and adaptive resistance.

### ROS modulators

ROS modulation supports two complementary therapeutic logics: increasing ROS to exceed tumor antioxidant capacity and induce oxidative-stress–mediated cell death, and modulating mitochondrial respiration to alter oxygen consumption and treatment sensitivity in metabolically heterogeneous tumors [68].

A pancreatic cancer synthesis emphasizes that increasing intracellular ROS is a promising strategy to make cancer cells more vulnerable to oxidative stress–induced cell death, supporting interest in pro-oxidant approaches while implying a need for careful evaluation of host toxicity in clinically complex settings [52].

MitoQ provides an illustrative example of a mitochondria-targeted antioxidant leveraged for oxygen-consumption modulation: solid tumors are described as metabolically heterogeneous, and MitoQ is reported to dose-dependently reduce cancer cell respiration, with preclinical evidence of radiosensitization in MDA-MB-231 tumors but not MCF7 cells, emphasizing context dependence and the need for predictive frameworks. MitoQ is also reported in preclinical breast cancer models to prevent local recurrence and metastatic dissemination at nanomolar concentrations that spare nonmalignant cells, and a robust response signature has been defined for use in bulk primary tumor biopsies, supporting biomarker-enriched development strategies [54].

### Mitochondrial genome editing

Mitochondrial genome editing is advancing at the level of tool development, with reports describing engineered base editors with enhanced activity and specificity and improved DdCBE and TALED variants used to model disease-associated mtDNA mutations and generate edited human cells with pathogenic phenotypes in modeling settings. Engineering approaches including high-fidelity mutations and nuclear export signal designs are described to reduce off-target editing in nuclear and mitochondrial genomes, and engineered TALED variants are reported to reduce RNA off-target edits by >99% while also minimizing off-target mtDNA mutations and bystander edits at target sites, supporting improved fidelity in preclinical systems [69, 70]. Despite these advances, mitochondrial genome editing remains primarily a preclinical approach, and challenges related to delivery, editing efficiency, heteroplasmy, off-target effects, and tissue specificity will need to be addressed before clinical translation in oncology.

### Next-generation immunotherapies

Mitochondrial biology is also being explored as a potential target for next-generation immunotherapeutic approaches. Within the evidence base summarized here, early clinical investigation includes an engineered mitochondrial vaccine study in patients with advanced solid tumors. ClinicalTrials.gov registry information for this study describes dose-escalation cohorts and objective response rate endpoints; however, clinical outcomes were not reported in the evidence available for this review [71]. These findings should therefore be considered preliminary and investigational, while further studies are needed to establish the safety, immunogenicity, clinical efficacy, and mechanisms underlying mitochondria-directed immunotherapeutic strategies.

### Clinical integration

Clinical integration of mitochondrial targeting into surgical oncology requires explicit recognition that tumors exhibit extensive metabolic heterogeneity and that cells can switch between glycolysis and OXPHOS or occupy hybrid states under environmental and therapeutic pressures, which can blunt single-pathway interventions and influence resistance biology across multimodal care.

### Neoadjuvant setting

A neoadjuvant “metabolic window” concept is supported by the observation that metabolic phenotyping strategies are proposed to classify tumors by metabolic phenotype to therapeutically target tumor-specific vulnerabilities, and by the existence of gene-signature approaches to quantify glycolysis versus OXPHOS activity using AMPK and HIF-1 downstream genes. Such approaches are aligned with evidence that cancer cells can access hybrid metabolic states and can switch between glycolysis and OXPHOS, implying that short pre-treatment windows may be used to evaluate pharmacodynamic shifts and early adaptation signals rather than only long-term outcomes [11, 72].

### Adjuvant setting

In the adjuvant setting, MA.32 provides strong evidence that adding metformin to standard breast cancer therapy did not improve invasive disease–free survival in unselected nondiabetic early breast cancer populations and increased grade 3 nonhematological adverse events and non-adherence, supporting the conclusion that broad empiric adjuvant deployment is not justified without a predictive framework and feasibility planning [57].

For apoptosis sensitization, venetoclax provides proof of principle for mitochondrial apoptosis targeting in hematologic malignancies, but effectiveness in solid tumors remains under investigation, reinforcing that adjuvant translation in resected solid tumors requires selection and combination logic rather than direct extrapolation [44].

### Peri-treatment feasibility

Across mitochondria-targeted strategies, feasibility constraints relevant to surgical pathways are illustrated by (i) toxicity and adherence limitations in large adjuvant metabolic trials and (ii) narrow therapeutic index and dose-limiting toxicities that can halt complex I inhibitor programs. These constraints align with broader observations that metabolic plasticity is a hallmark relevant to tumor resistance, implying that therapeutic strategies need to anticipate adaptation and evaluate early pharmacodynamic readouts rather than relying only on baseline assumptions about metabolic dependence [4, 60].

### Patient stratification

Patient stratification is central because heterogeneity occurs at the level of tumor type, tumor subtype, within the tumor itself, and within the tumor microenvironment, and because glucose utilization and carbon sources are described as more heterogeneous than initially appreciated. Classification of tumors by metabolic phenotype is explicitly proposed as a way to therapeutically target tumor-specific vulnerabilities, which implies that translational surgical oncology programs should prioritize feasible phenotype classification tools and response biomarkers [73, 74].

The Table 2 summarizes mitochondrial and metabolism-adjacent biomarkers and readouts explicitly supported by the cited sources that may have utility for risk stratification, therapy selection, or pharmacodynamic monitoring.

**TABLE 2:** Mitochondrial and metabolism-associated biomarkers for cancer stratification and therapeutic monitoring.

Biomarker or readout	Cancer context	Proposed translational value
PGC-1α-dependent mitochondrial oxidative metabolism	Subset of human melanoma	Defines an OXPHOS-dependent subtype concept relevant to stratification for OXPHOS-focused strategies
High mitochondrial mass	Breast cancer model CSCs (MCF7)	Proposed metabolic biomarker for anabolic CSC populations relevant to identifying therapy-resistant subpopulations in translational work
AMPK and HIF-1 downstream gene signatures	Pan-cancer and systems-level analyses	Proposed quantification of glycolysis versus OXPHOS activity to support metabolic phenotyping and hybrid-state inference
mtDNA vulnerability to oxidative damage	General tumor biology	Mechanistic rationale supporting mtDNA-linked instability and consideration of mtDNA-centered biomarkers in oxidative stress–rich contexts
mtDNA:gDNA ratio increase during therapy	AML and solid tumors treated with IACS-010759	Potential pharmacodynamic marker interpreted as rapid adaptation to complex I inhibition in phase I analyses
CA 15-3 change	MA.32 breast cancer trial	Pharmacodynamic biomarker modulation with metformin that may support mechanism-focused window endpoints despite negative survival outcomes
Estradiol change	MA.32 breast cancer trial	Systemic biomarker modulation with metformin relevant to estrogen-sensitive cancer biology hypotheses in translational studies
MitoQ response signature	Preclinical breast cancer models	A robust response signature defined for bulk primary tumor biopsies to support future trial stratification and monitoring

## Discussion

### Challenges and future directions

Clinical trajectories of mitochondria-targeted therapies emphasize that mechanistic plausibility and preclinical signals do not guarantee a favorable therapeutic index in humans, particularly for systemically delivered OXPHOS inhibitors. MA.32 demonstrates a negative adjuvant outcome for metformin in unselected early breast cancer and shows increased grade 3 nonhematological adverse events and increased non-adherence with metformin, highlighting both efficacy and feasibility barriers for broad repurposing strategies in surgical populations. IACS-010759 phase I trials similarly demonstrate a direct barrier for systemic complex I inhibition, with narrow therapeutic index, dose-limiting lactate elevation and neurotoxicity, no recommended phase 2 dose, modest target inhibition, limited antitumor activity, and trial discontinuation, underscoring the need for safer delivery approaches, combination strategies, or stronger biomarker selection [75].

For apoptosis targeting, venetoclax demonstrates the power of Bcl-2 family modulation in AML, but solid tumor effectiveness remains under investigation, and monotherapy is described as limited, reinforcing that solid tumor translation requires context-specific dependency mapping and combination regimens rather than direct transplantation of hematologic paradigms [76].

A major research gap is the development of practical approaches to characterize metabolic heterogeneity within individual tumors [7, 77, 78]. Bulk metabolic measurements may not adequately capture differences between tumor regions or between malignant and stromal cell populations. Emerging spatial technologies such as mass spectrometry imaging (MSI) may help address this limitation by mapping metabolites and metabolic features within anatomically defined regions of surgical tumor specimens. For example, tumor core, invasive margin, hypoxic or necrotic areas, and adjacent nonmalignant tissue could be analyzed separately to determine whether distinct metabolic profiles are associated with different tumor regions.

MSI-based metabolic information could then be integrated with single-cell sequencing or spatial transcriptomic analysis from matched surgical specimens. Single-cell sequencing may help identify cellular populations associated with distinct metabolic programs, including malignant cells, stromal cells, macrophages, T cells, and other immune populations, while spatial transcriptomic approaches can preserve their location within the tumor microenvironment [79]. Integration of these datasets may help determine whether glycolysis-dominant, OXPHOS-dominant, or hybrid metabolic states are associated with specific tumor-cell populations or microenvironmental regions [80].

A practical approach would therefore be to collect and spatially annotate surgical specimens, perform MSI to characterize regional metabolic features, and use matched single-cell or spatial transcriptomic profiling to define the cellular populations associated with these metabolic states. These data could then be integrated with histopathological findings, treatment exposure, and clinical outcomes to identify metabolic features associated with therapeutic response, resistance, or recurrence. Such approaches may also help identify metabolically plastic tumor populations that switch between glycolysis and OXPHOS following treatment and may therefore contribute to therapeutic resistance [81].

In parallel, mitochondrial genome editing tool development is accelerating and shows improved activity and fidelity with major reductions in RNA off-target edits, but these advances are described in the context of mitochondrial genome study and disease modeling, implying that cancer therapy translation will require additional work on safety, feasibility, and clinically relevant delivery and selection frameworks [82]. Future studies should therefore integrate metabolic phenotyping, spatial analysis, and functional mitochondrial measurements with clinical outcomes rather than relying on a single metabolic marker.

The timing of metabolic interventions will also be important in the postoperative setting. Because the early response to surgery requires mobilization of energy and substrates for inflammation and tissue repair, broad inhibition of mitochondrial metabolism during this period could potentially interfere with the normal transition from a catabolic to an anabolic state. Therefore, mitochondrial-targeted therapies may need to be carefully timed around surgery and evaluated together with nutritional status, wound healing, and recovery before they are incorporated into perioperative treatment strategies.

### Conclusion

This review reframes mitochondrial dysfunction for a surgical oncology readership by emphasizing that mitochondrial metabolism is active and necessary for tumor growth and supports tumor anabolism, and that mitochondria and mtDNAs are described as essential for cancer cell growth with mtDNA mutations altering metabolism and adaptation across cancers. A core translational implication is that tumor metabolism is heterogeneous and flexible, with dual glycolysis/OXPHOS capacity and hybrid states described as supporting metabolic plasticity that is associated with metastasis and therapy resistance, motivating phenotype-aware biomarker development and treatment selection strategies in multimodal cancer care.

Clinical trial lessons show that broad empiric mitochondria-targeted approaches can fail on efficacy and feasibility grounds: MA.32 shows no invasive disease–free survival benefit for adjuvant metformin and highlights increased grade 3 toxicity and non-adherence, while IACS-010759 phase I results demonstrate a narrow therapeutic index with dose-limiting toxicities, inability to establish an RP2D, limited activity, and discontinuation. These experiences reinforce that biomarker-driven patient selection and combination strategies are essential, consistent with analyses supporting metabolic phenotype classification to target tumor-specific vulnerabilities and with evidence that OXPHOS inhibitors have limited efficiency in monotherapy and may be most valuable in combinations.

Near-term progress is most likely to come from integrating metabolic phenotyping tools (including signatures that quantify glycolysis versus OXPHOS), developing robust pharmacodynamic and response signatures (e.g., for MitoQ), and aligning mitochondria-targeted interventions with clinically feasible endpoints and safety requirements relevant to surgical and peri-treatment care pathways.
